# Synovial fluid level of aggrecan ARGS fragments is a more sensitive marker of joint disease than glycosaminoglycan or aggrecan levels: a cross-sectional study

**DOI:** 10.1186/ar2735

**Published:** 2009-06-22

**Authors:** Staffan Larsson, L Stefan Lohmander, André Struglics

**Affiliations:** 1 Department of Orthopaedics, Clinical Sciences Lund, Lund University, SE-221 85 Lund, Sweden

## Abstract

**Introduction:**

Aggrecanase cleavage at the ^392^Glu-^393^Ala bond in the interglobular domain (IGD) of aggrecan, releasing N-terminal ^393^ARGS fragments, is an early key event in arthritis and joint injuries. Here, we use a quantitative immunoassay of aggrecan ARGS neoepitope fragments in human synovial fluid to determine if this cleavage-site specific method better identifies joint pathology than previously available less specific aggrecan assays.

**Methods:**

Synovial fluid (SF) from 26 people with healthy knees (reference) and 269 patients were analyzed in a cross-sectional study. Patient groups were acute inflammatory arthritis, acute knee injury, chronic knee injury and knee osteoarthritis (OA). Aggrecan ARGS fragments were assayed by ELISA using the monoclonal antibody OA-1. Total aggrecan content was analyzed by an ELISA using the monoclonal antibody 1-F21, and sulfated glycosaminoglycan by Alcian blue precipitation.

**Results:**

Aggrecan ARGS fragment concentrations in all groups differed from the reference group (*P *< 0.001). The acute inflammatory arthritis group had the highest median level, 177-fold greater than that of the reference group. Median levels (in pmol ARGS/ml SF) were: reference 0.5, acute inflammatory arthritis 88.5, acute knee injury 53.9, chronic knee injury 0.5 and OA 4.6. In contrast, aggrecan and sulfated glycosaminoglycan concentrations varied much less between groups, and only acute inflammatory arthritis and acute knee injury were found to have a two-fold increase in median levels compared to the reference.

**Conclusions:**

Levels of aggrecan ARGS fragments in human synovial fluid are increased in human arthritis, OA and after knee injury, likely reflecting an enhanced cleavage at the ^392^Glu-^393^Ala bond in the IGD by aggrecanase. An assay that specifically quantified these fragments better distinguished samples from joints with pathology than assays monitoring aggrecan or glycosaminoglycan concentrations. The newly developed ARGS fragment assay can be used to monitor aggrecanase activity in human joint disease and experimental models.

## Introduction

Proteolysis of aggrecan is an early and critical feature of cartilage degradation in arthritis and after knee injury, and is measurable as an elevation of aggrecan release from the cartilage into the synovial fluid (SF) [1-4]. Although proteases, such as matrix metalloproteases (MMPs), cathepsins and calpains, are involved [5], aggrecanase plays a major role in aggrecan degradation in murine [6,7] and human [4,8-15] joint disease. There are five known aggrecanase cleavage sites in aggrecan [16]. The most severe aggrecanase cleavage in terms of destructive loss of sulfated glycosaminoglycan (sGAG) from the tissue, is at the ^392^Glu-^393^Ala bond in the interglobular (IGD) domain of aggrecan, releasing N-terminal ^393^ARGS neoepitope fragments.

ARGS neoepitope aggrecan fragments released into the SF, as detected by western blot or amino acid sequencing, have been associated with joint diseases [4,8,9,17,18] and have also been detected as a result of normal turnover [4,17]. When quantified by a western blot method, the proportion of aggrecan in SF having the neoepitope ARGS was elevated in arthritis and joint injury compared with individuals with healthy knees [4]. Fragments carrying the same neoepitope were also found in serum from patients with rheumatoid arthritis, but not in healthy controls [15].

Results from several ELISAs have been presented that measure levels of aggrecan neoepitopes in medium from human cartilage explants [10,11,13,19]. By measuring neoepitope concentrations, aggrecanase cleavage at the ^392^Glu-^393^Ala bond has been confirmed as a major contributor to aggrecan loss from cartilage stimulated by cytokines [10,11,13-15]. However, with the exception of small-scale quantitative western blots [4], only assays of non-specific aggrecan fragments [1,20], of newly synthesized aggrecan bearing the 846 epitope [21] or of sGAG [22] have been reported in studies of human SF.

In this cross-sectional study, comparing people with healthy knees with those with acute inflammatory arthritis, acute knee injury, chronic knee injury, or knee osteoarthritis (OA), we quantified the SF levels of the aggrecan ARGS neoepitope with a modified sandwich ELISA [19], and compared it with aggrecan assays not specific for this neoepitope. We hypothesized that ARGS neoepitope concentrations in SF would differ between these groups and be a more sensitive measure of joint disease than previously used aggrecan or sGAG assays.

## Materials and methods

### Amino acid numbering

All amino acid numbering of aggrecan is herein based on full-length human aggrecan, accession number [Swiss-Prot:P16112], starting with the N-terminal ^1^MTTL-amino acid sequence.

### Materials

Alcian blue 8GS (C.I. 742240) was from Chroma-Gesellschaft (Köningen, Germany). 4-(2-aminoethyl)-benzenesulfonyl fluoride (AEBSF), 6-aminohexonic acid (EACA), benzamidine-HCl, BSA, chondroitin sulfate type C from shark cartilage (no. C4384), ethylenediaminetetra acetic acid (EDTA), N-ethylmaleimide (NEM), 2-(N-morpholino) ethanesulfonic acid (MES), phenylmethylsulfonyl fluoride (PMSF), and phosphate buffered saline with TWEEN (PBST) buffer (0.01 M sodium phosphate, 0.138 M sodium chloride, 0.0027 M potassium chloride, 0.05% TWEEN 20; pH 7.4) were from Sigma (St. Louis, MO, USA). Cesium chloride and guanidinium hydrochloride were from Merck (Darmstadt, Germany). Molecular weight markers 10 to 250 kDa (no. 161-0373) were from BioRad (Hercules, CA, USA). Human recombinant ADAMTS-4 (a disintegrin and metalloproteinase with thrombospondin motifs, aggrecanase-1) was from GlaxoSmithKline (Collegeville, PA, USA) [23]. ECL Plus detection was from Amersham Biosciences (Buckinghamshire, UK). Polyvinylidene difluoride (PVDF) membranes, Tris-acetate mini gels (3 to 8%), LDS sample buffer, Tris-acetate SDS running buffer and transfer buffer were from Invitrogen (Carlsbad, CA, USA). Non-fat dry milk by Semper (Sundbyberg, Sweden) was from the local supermarket.

Quick-Seal centrifuge tubes (2 ml no. 344625, 12.5 ml no. 342413), tube sealer (no. 342428), tube slicer (no. 303811) were from Beckman Coulter (Palo Alto, CA, USA). The monoclonal antibody (MAb) OA-1, with or without biotinylation, recognizing the neoepitope sequence ARGSVIL (representing the N-terminus of human aggrecan cleaved between ^392^Glu and ^393^Ala in the interglobular domain) was kindly provided by Michael Pratta (GlaxoSmithKline, Collegeville, PA, USA) [19]. Tetramethylbenzidine (TMB)-hydrogen peroxidase (H_2_O_2_) solution (no. 50-76-00) and peroxidase labeled streptavidin (no. 14-30-00) were from KPL (Gaithersburg, MD, USA). Hyaluronidase from *Streptomyces hyalurolyticus *(EC 4.2.2.1), chondroitinase ABC protease free (EC 4.2.2.4), keratanase (EC 3.2.1.103) and keratanase II (from *Bacilus *species Ks 36) were from Seikagaku (Tokyo, Japan). Keratan sulfate (KS) capture 96-well plates (no. 42.146.08) were from Biosource International (Camiro, CA, USA).

### Subjects and samples

Knee SF from 26 knee healthy volunteers and 269 patients were obtained from a cross-sectional convenience cohort, where each individual, after informed consent, provided a sample at one time point only. Diagnosis was made by arthroscopy, radiography, assessment of SF and clinical examination [1]. Samples were centrifuged at 3000 g and aliquots of the supernatant were stored at -80°C. All patient-related procedures were approved by the ethics review committee of the Medical Faculty of Lund University.

Diagnostic groups were healthy knee references (REF), acute inflammatory knee arthritis (AA), knee OA, and injured knee (anterior cruciate ligament rupture and/or meniscus tear) grouped as acute knee injury (AI; 0 to 12 weeks after injury) or chronic knee injury (CI; > 12 weeks after injury; Table 1). Joint changes, assessed by arthroscopy and radiography, were scored ranging from 1 to 10, where 1 represents a normal joint by arthroscopy and radiography; 2 to 5 represents an increasing extent and severity of fibrillation and clefts in the joint cartilage by arthroscopy in joints appearing normal on radiographs; and 6 to 10 represents increasing degrees of radiographic joint space narrowing consistent with OA [24]. Thirty-one samples lacked arthroscopic and/or radiographic data needed for assessment of OA score.

**Table 1:** Characteristics of the study patients and reference group

					OA score	
					
Study group	Subject number	Male, %	Age, years	Time of sampling, weeks after injury or onset		no.
REF	26	62	27 (17 to 89)	-	1 (1 to 1)	16
AA	48	60	66 (30 to 92)	0.4 (0 to 510)	7 (3 to 9)	31
AI	69	81	27 (16 to 59)	1.4 (0 to 11.9)	1 (1 to 5)	67
CI	123	77	40 (16 to 70)	61 (12.7 to 1926)	2 (1 to 8)	121
OA	29	66	61 (25 to 92)	125 (0 to 772)	7 (2 to 9)	29

Age, time of sampling and OA score in median values (range).

REF = healthy knee reference; AA = acute inflammatory arthritis (46 acute pyrophosphate arthritis/pseudogout, one rheumatoid arthritis and one acute reactive arthritis/Yersinia); AI = acute knee injury (47 anterior cruciate ligament ruptures and one posterior, with or without meniscus tear and meniscus tear alone, 0 to 12 weeks after injury); CI = chronic knee injury (120 anterior cruciate ligament ruptures and three posterior, with or without meniscus tear and meniscus tear alone, > 12 weeks after injury); OA = knee osteoarthritis. The OA score ranges from 1 to 10 where 1 represents a normal joint; see Materials and Methods for a detailed description.

To study injury-dependent aggrecan fragment release at different times after injury, these samples were grouped as meniscal injury alone (MEN) or cruciate ligament rupture with or without an associated meniscus injury (ACL), stratified by time after injury (0 to 4, 4 to 12, 12 to 26, 26 to 52, or > 52 weeks).

Patient samples were selected from a biobank by one of the authors (LSL) on the basis of clinical diagnosis, without reference to any previously available assay data.

### Cartilage aggrecan digest as ARGS standard

From the pool of human knee OA cartilage (10 patients) proteoglycans were extracted with guanidinium hydrochloride (4 M) in the presence of proteinase inhibitors (10 mM EDTA, 100 mM EACA, 10 mM NEM, 5 mM benzamidine-HCl and 5 mM PMSF) and aggrecan was then isolated by associative-dissociative cesium chloride density gradient centrifugation in the presence of the proteinase inhibitors [25]. Fraction A1D1 was collected and dialyzed against Millipore-water prior to freeze drying [18]. As described, this fraction contains only large aggrecan fragments, containing the IGD, without G1-IPEN and G1-TEGE fragments [18]. Human aggrecan monomers were quantified based on dry weight assuming a molecular weight of 1.5 × 10^6 ^g/mol.

Full-length human recombinant ADAMTS-4 was cloned, expressed, and purified at GlaxoSmithKline (Collegeville, PA, USA) [23]. ADAMTS-4 (3.1 nM) was incubated with the A1D1 fraction of human aggrecan (346 nM) for 30 hours at 37°C in 50 mM Tris-HCl, 100 mM sodium chloride (NaCl), 10 mM calcium chloride (CaCl_2_), pH 7.5, achieving complete conversion of the G1-containing starting material to G1-TEGE fragments and the corresponding ARGS fragments. The digest was quenched with 25 mM EDTA and monitored for complete digestion by G1, TEGE, and ARGS western blots (data not shown). The digest was used as an ARGS standard in the aggrecan ARGS ELISA.

### Aggrecan ARGS ELISA

Quantification in SF of aggrecan fragments with the N-terminal ^393^ARGS was by a sandwich ELISA using an anti-KS antibody as capture and the monoclonal neoepitope antibody OA-1 for detection of specific fragments [19]. After modification for use in SF, the assay was conducted as follows:

#### Sample treatment

ARGS standard (ADAMTS-4 digested cartilage A1D1 aggrecan) was treated with chondroitinase ABC as described [18]. SF samples were digested with hyaluronidase (0.01 turbidity reducing unit/μl SF for three hours at 60°C in 50 mM sodium acetate, 10 mM EDTA, 0.25 mM AEBSF, pH 6), treated with chondroitinase ABC (0.8 mU/μl SF for 30 minutes at 37°C in 50 mM Tris-acetate, 75 mM sodium acetate, 15 mM EDTA, 0.125 mM AEBSF, pH 7.6), boiled in a water bath for five minutes, and spun (12,500 g, five minutes) collecting the supernatant.

#### ELISA

Duplicates of 300 μl of ARGS standards (ADAMTS-4 digested cartilage A1D1 aggrecan; 0.02 to 1 nM ARGS) or supernatant of boiled and spun SF samples (final SF dilution 1:50 to 1:6400) were incubated in the presence of 1% w/v BSA, 20 mM MES, 150 mM NaCl, pH 5.3 on KS capture plates coated with an anti-KS antibody (Biosource International, Camiro, CA, USA) over night at 4°C on a plate shaker. Following washes (6 × 400 μl PBST), plates were incubated with biotinylated MAb OA-1 (150 μl/well, 1.5 μg/ml in PBST with 0.1% w/v non-fat dry milk) for two hours at 37°C on a plate shaker. Plates were washed (as above) and incubated with horseradish peroxidase-conjugated streptavidin (150 μl/well, 1 μg/ml in PBST) for one hour at room temperature on a plate shaker. Following a wash, a five-minute incubation of TMB-H_2_O_2 _solution (150 μl/well) and acidification with 1 M phosphoric acid (150 μl/well), absorbance at 450 nm was measured spectrophotometrically using a Multiscan Multisoft plate reader (Labsystems, Helsinki, Finland) and the software Ascent 2.4.2 (Thermo Electron, Waltham, WA, USA).

#### Spiking

SF from individuals with ARGS concentrations suited for analysis diluted at 1:50, 1:400, 1:800, and 1:1600 were spiked with equimolar concentrations of ARGS standard (ADAMTS-4-digested cartilage A1D1 aggrecan) and analyzed in the ARGS ELISA.

ARGS neoepitope assays were performed with no knowledge of clinical diagnosis or previous assay data.

### Aggrecan and sGAG quantification in synovial fluid

Aggrecan content was analyzed by a slightly modified competition ELISA using the mAb 1-F21 recognizing a protein sequence within or close to the KS domain [20,26]. The 1-F21 ELISA differed from the original [20] as follows: concentration of chondroitinase-digested A1D1 was 1.25 μg/ml when coating; all washes were 3 × 200 μl; plates were blocked after coating (1% BSA, 200 μl/well, 30 minutes at room temperature); the primary antibody 1-F21 was diluted to 1:10,000; the secondary antibody (Dakopat nr. P447) was diluted to 1:2000.

Concentration of sGAG was measured by Alcian blue precipitation modified from Björnsson [22]. Samples and chondroitin sulfate standards (25 μl) were precipitated for two hours at 4°C with 0.04% w/v Alcian blue, 0.72 M guanidinium hydrochloride, 0.25% w/v Triton X-100, and 0.1% v/v H_2_SO_4 _(0.45 ml). The precipitates were collected after centrifugation (16,000 g, 15 minutes, 4°C), then dissolved in 4 M guanidinium hydrochloride, 33% v/v 1-propanol (0.25 ml), and transferred to 96-well micro-titer plates prior to absorbance measurement at 600 nm.

These data were available from previous studies using these samples [26-28].

For molar comparison of ARGS fragments and aggrecan, conversion from microgram sGAG/ml to pmol aggrecan/ml was made assuming an average aggrecan molecular weight of 1.5 × 10^6 ^g/mol and assuming that 75% of this weight was sGAG [4].

### Western blot

Aggrecan fragments captured in the ARGS ELISA by the anti-KS antibodies were analyzed by western blot. Following a completed ARGS ELISA, plates were washed with PBST and incubated with 4 M guanidinium hydrochloride (150 μl/well) for 30 minutes at room temperature on a plate shaker. To obtain enough material for western blot analysis, the well contents of standard wells (74 wells) and wells of SF from 152 patients (152 wells) were pooled separately and dialyzed in 10,000 kDa cut-off dialysis cassettes (Slide-A-Lyzer, Pierce, Rockford, IL, USA) against Millipore water containing protease inhibitors [18]. Samples were freeze dried, dissolved in deglycosylation buffer and digested by chondroitinase, keratanase and keratanase II [18]. Samples were precipitated in ice-cold acetone, and pellets were dissolved in two times concentrated sample buffer.

ADAMTS-4 digested aggrecan (used in the ELISA as standard) and a D1 fraction of pooled SF from 40 OA patients were chondroitinase, keratanase and keratanase II digested [18].

All samples were run on a 3 to 8% Tris-acetate SDS-PAGE gel, transferred to a PVDF membrane and ARGS fragments were visualized using the MAb OA-1 [18].

### Western blot quantification

Quantification of ARGS fragment in SF by western blot was performed as described [4] using the same mAb for detection (OA-1) and the same standard as in the ARGS ELISA.

### Statistical analysis

For some patients the available volume of SF was not large enough to perform all assays, which explains the variation in numbers between assays. Of the 295 subjects, 113 had ARGS fragment values below the level of detection (i.e. < 1 pmol ARGS/ml SF). Each was assigned a value of 0.5 pmol ARGS/ml, or half the lower limit of detection. To assess differences among the study groups, either a two-tailed Mann-Whitney U rank sum test with Bonferroni correction was used after Kruskal-Wallis testing, or a Chi-squared test, as appropriate. For correlation analysis Spearman's rank order correlation (r_S_) was used. *P *values below 0.05 were considered significant unless otherwise noted. Statistical calculations were performed using Statistical Package for the Social Sciences (SPSS, Chicago, IL, USA) for Windows version 15.0.

## Results

### Technical performance of the ARGS ELISA

SF samples needed to be diluted 1:50 or more for a linear recovery at different dilutions; at dilutions below 1:50 the signal was reduced due to unknown matrix effects (results not shown). With a linear measuring range for the standards of 0.02 to 1 pmol ARGS/ml, and a minimal dilution of SF of 1:50, the lower limit of detection was then recalculated to undiluted SF 1 pmol ARGS/ml SF. Intra assay coefficient of variation (CV) was 6% (n = 10), the inter assay CV for the two groups of KS capture plates used were 12% (n = 5) and 16% (n = 23), respectively, and the total inter assay CV for the control SF sample included on all plates was 20% (n = 28; Table 2). The mean spiking recovery at dilutions 1:50 to 1:1600 was 99% (range 75 to 121%; Table 2).

**Table 2:** Technical performance of the KS capture OA-1 ARGS ELISA

Linear measuring range of standard	0.02 to 1 pmol/ml			
Minimal dilution of SF	1:50			
Minimal detectable concentration in neat SF	1 pmol/ml SF			
Intra assay CV (n = 10)	6.1%			
*Inter assay, intra lot CV (n = 5, 1^st ^lot)	12.2%			
*Inter assay, intra lot CV (n = 23, 2^nd ^lot)	15.6%			
Inter assay, inter lot CV (n = 28)	19.7%			
Dilution of SF	1:50	1:400	1:800	1:1600
Spiking recovery (mean; range)	116%; 109 to 121%	93%; 75 to 104%	81%; 75 to 88%	104%; 98 to 113%

* Between assay variation in lot numbers (1^st^, #5L21/1; 2^nd^, #7F25/1) of the KS capture plates from Biosource.

CV = coefficient of variation; KS = keratan sulfate; SF = synovial fluid.

**Figure 1:**
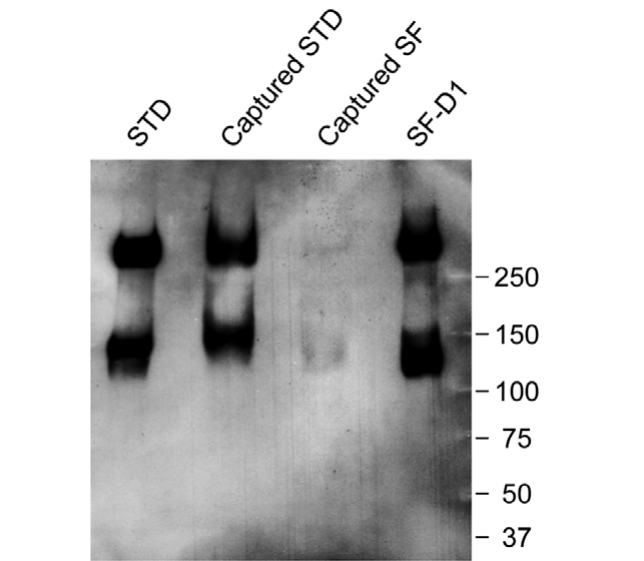
Anti-ARGS western blot of ELISA-captured material. Aggrecan fragments captured by the anti-keratan sulfate (KS)-coated plates were extracted after a completed ELISA and analyzed by western blot. Seventy-four wells of captured ARGS standards (STD) and 152 wells of SF from 152 patients were used. The samples were chondroitinase, keratanase, and keratanase II digested, separated on a SDS-PAGE gel, transferred to a polyvinylidene difluoride (PVDF) membrane and probed with the ARGS antibody OA-1. For comparison, the STD (0.5 μg sulfated glycosaminoglycan (sGAG)/well) and an SF D1 sample pooled from 40 osteoarthritis (OA) patients (0.75 μg sGAG/well) were used as controls. The size (kDa) and position of the molecular weight markers are indicated.

Anti-ARGS western blot analysis of aggrecan fragments captured by the ELISA plates, showed that the ARGS fragments present in the standard were also captured by the anti-KS plates (Figure 1). The SF ARGS fragments captured by the plates showed the same fragment pattern as those detected in an SF D1 control sample and in the two standard samples.

### Aggrecan, sGAG, and ARGS fragment concentrations in synovial fluid

**Figure 2:**
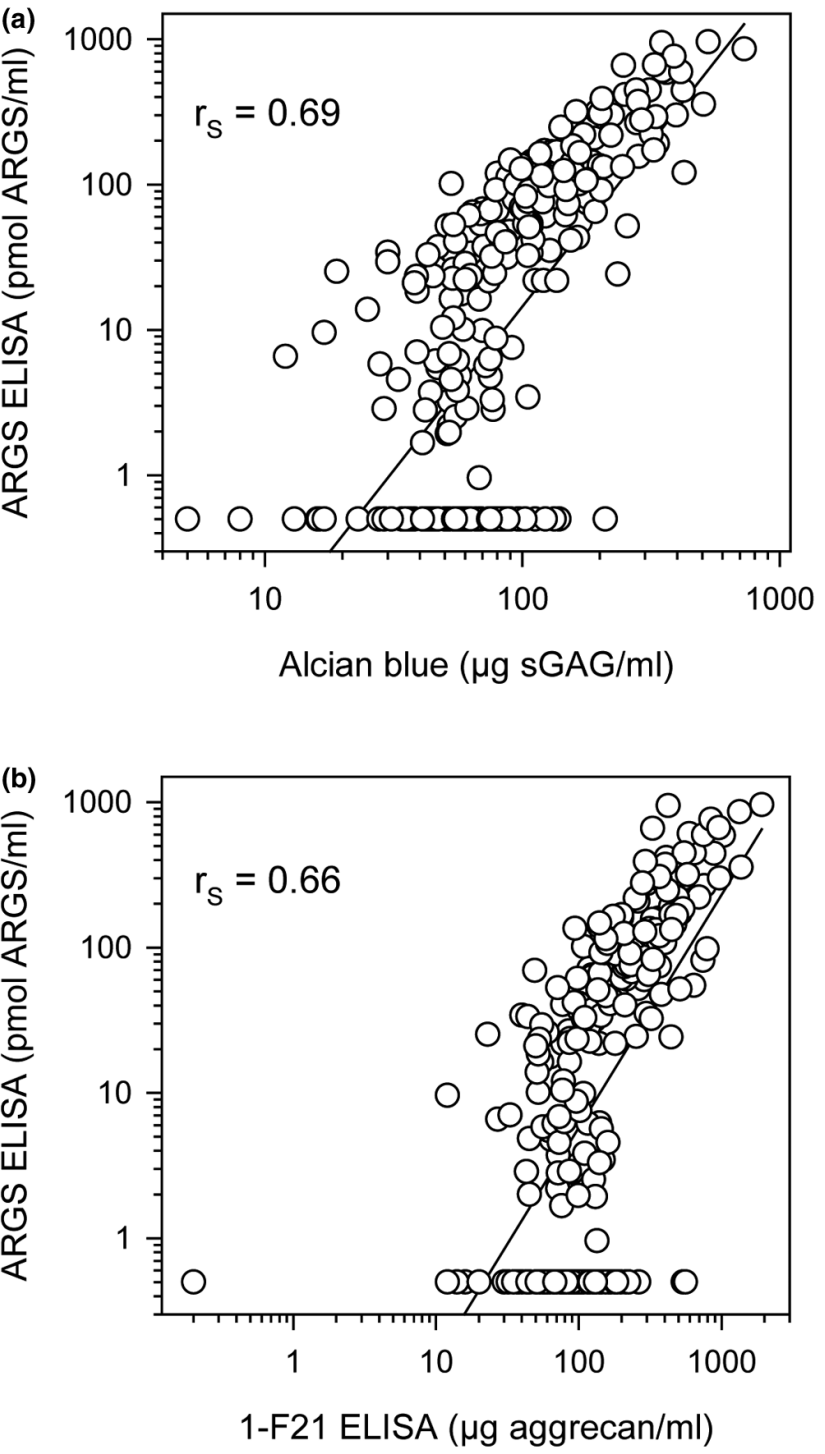
Regression analysis of aggrecan fragment data. The same samples of synovial fluid were analyzed by three different assays (see Material and Methods for details). Concentration of aggrecan fragments carrying the neoepitope ARGS by ELISA versus **(a) **sGAG concentration by Alcian blue precipitation (n = 293) and versus **(b) **aggrecan concentration by 1-F21 ELISA (n = 285). Solid lines show the first-order regression. Note the logarithmic X- and Y-axes. Spearman's rank order correlations (r_S_) are given for each relationship with *P *< 0.0001.

The concentrations of aggrecan measured as 1-F21 reactivity, sGAG, and aggrecan fragments bearing the ARGS neoepitope are summarized in Table 3. As shown [26], there was a strong correlation between aggrecan fragment concentration measured by the 1-F21 ELISA and the concentration of sGAG (r_S _= 0.82, data not shown). The ARGS concentration showed a more moderate correlation with the concentrations of sGAG (r_S _= 0.69; Figure 2a) and aggrecan (r_S _= 0.66; Figure 2b).

**Table 3:** Synovial fluid aggrecan fragment data in all subjects

	OA-1 ARGS ELISA (pmol ARGS/ml)	Alcian blue precipitation (μg sGAG/ml)	1-F21 ELISA (μg aggrecan/ml)
n	295	293	285
Mean	75	104	199
Median	10	74	120
Range	0.5 to 961	5 to 728	0.2 to 1912

To validate the identity of the fragments responsible for the signal below the detection limit of the ARGS ELISA, 32 samples, of which 10 were below ELISA detection, were analyzed by western blot quantification [4], using the same mAb for detection, and compared with aggrecan fragment content as measured by sGAG. The molar proportion of aggrecan fragments bearing the ARGS neoepitope (i.e. ARGS/aggrecan) as measured by western blot was calculated. In the 10 samples below the detection limit of the ELISA, the median proportion of ARGS-bearing fragments out of aggrecan was 1.8% (range 1.2 to 6.4%) and in the samples above the detection limit, the median proportion was 23.8% (2.6 to 59.2%). Conversion from μg sGAG/ml to pmol aggrecan/ml is described in Material and Methods.

### Aggrecan ARGS fragments in diagnostic groups

**Figure 3:**
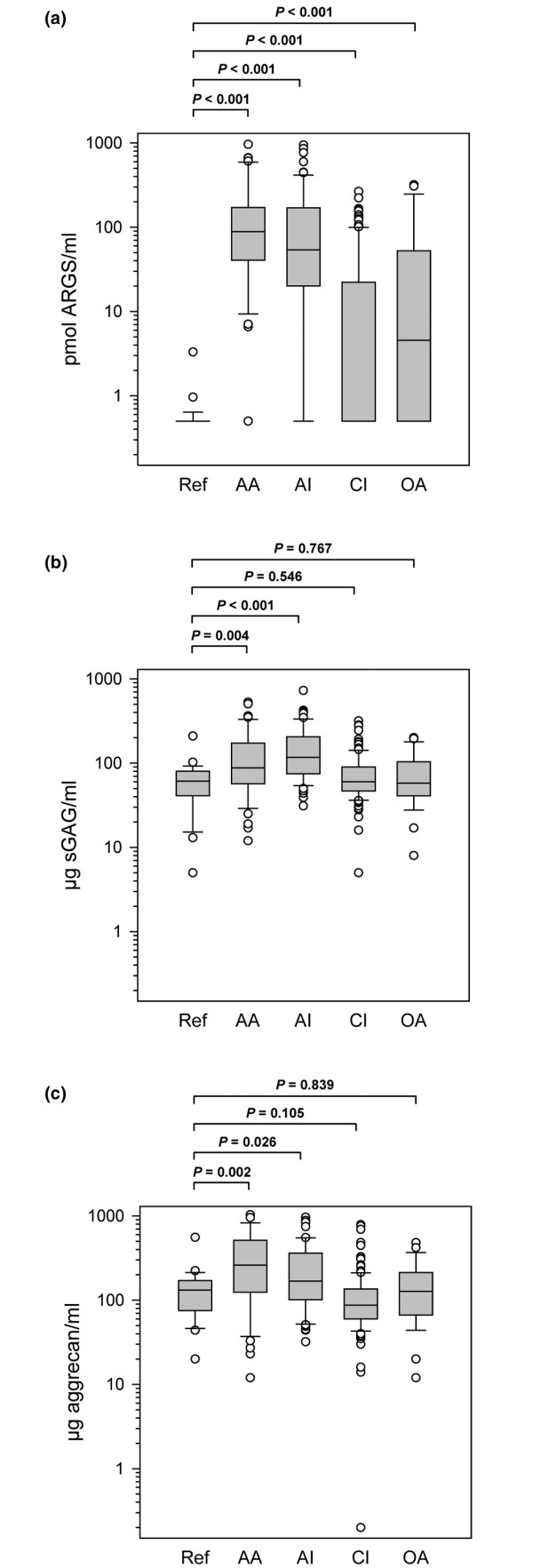
Aggrecan fragment concentrations in the study groups. Concentrations of **(a) **ARGS fragments, **(b) **sulfated glycosaminoglycan (sGAG), and **(c) **aggrecan in the study groups healthy knee reference (REF), acute inflammatory arthritis (AA), acute knee injury (AI), chronic knee injury (CI), and knee osteoarthritis (OA). The boxes define the 25^th ^and 75^th ^percentiles with a line at the median, error bars defining the 10^th ^and 90^th ^percentiles and circles represents individual outliers. Note that in panel (a) the median level of the chronic injury group is the same as the lower limit of the box; 0.5 pmol ARGS/ml. After Bonferroni correction, *P *values below 0.013 are considered significant to retain the 0.05 overall significance level.

Concentrations of aggrecan fragments carrying the neoepitope ARGS were elevated in all groups compared with the healthy knee reference group (Figure 3a). The median levels (in pmol ARGS/ml SF) were: REF 0.5 (range 0.5 to 3.3), AA 88.5 (0.5 to 961), AI 53.9 (0.5 to 946), CI 0.5 (0.5 to 266), and OA 4.6 (0.5 to 318). Similarly, all patient groups differed from the reference (*P *< 0.001) regarding the proportion of samples in each diagnostic group with ARGS concentration above the lower limit of detection (1 pmol ARGS/ml) as tested by Chi-squared tests. The percentages of detectable samples were 96% (AA), 87% (AI), 46% (CI), and 62% (OA) compared with 7.7% in REF. The sensitivity of ARGS fragment concentration as a marker for joint disease was 67% with a specificity of 92% (Table 4). We found no significant influence of age or sex on the SF levels of ARGS fragments (data not shown).

**Table 4:** Sensitivity and specificity of aggrecan fragment measurements

Assay	Cut-off	AUC	Sensitivity	Specificity
OA-1 ARGS ELISA	1 pmol ARGS/ml	82%	67%	92%
Alcian blue precipitation	88.5 μg sGAG/ml	63%	40%	92%
1-F21 ELISA	188.5 μg aggrecan/ml	53%	32%	91%
ARGS/sGAG*	5%	83%	65%	96%

*The molar proportion of aggrecan fragments in SF detected as ARGS neoepitope fragments, measured by Alcian blue precipitation and the ARGS ELISA respectively.

Sensitivity = the proportion of positives (diseased) correctly identified by the test.

Specificity = the proportion of negatives (healthy) correctly identified by the test.

AUC = area under receiver operating characteristic (ROC) curve.

Cut-offs were chosen based on ROC curve analysis where the sum of sensitivity and specificity was highest.

### sGAG and aggrecan in diagnostic groups

Median concentrations of sGAG were elevated only in the AA (*P *= 0.004) and AI groups (*P *< 0.001) compared with the REF group (Figure 3b). Similarly, concentrations of aggrecan measured by the 1-F21 ELISA were different from REF only in AA (*P *= 0.002) and AI (*P *= 0.026; Figure 3c). The sensitivities of sGAG and aggrecan fragment concentrations as markers for disease were 40% and 32% with specificities of 92% and 91%, respectively (Table 4). We found no significant influence of sex on the SF levels of sGAG or aggrecan (data not shown). However, age correlated negatively with sGAG concentration in the AA group (r_S _= -0.292) and aggrecan concentration in the AI and CI groups (r_S _= -0.335 and r_S _= -0.230 respectively).

### ARGS and aggrecan fragment release in relation to time after injury

**Figure 4:**
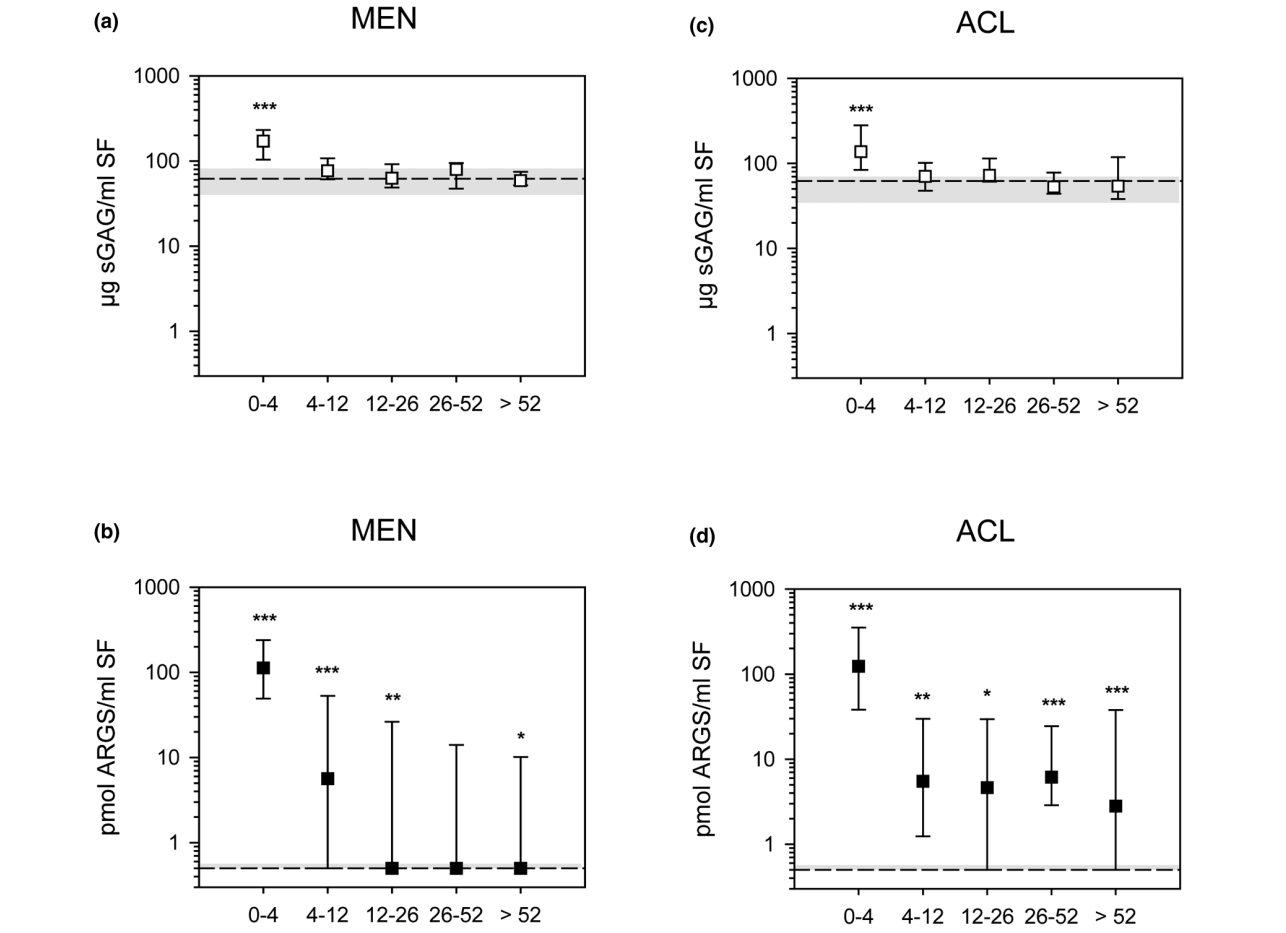
Aggrecan release after knee injury. Samples were ordered by time after knee injury (weeks) and by **(a, b**) meniscal injury alone (MEN) or **(c, d) **by anterior cruciate ligament rupture with or without an associated meniscus injury (ACL). Values are median concentrations of sulfated glycosaminoglycan (sGAG; open squares) and ARGS (filled squares) with 25^th ^and 75^th ^percentiles, compared with the medians (dashed lines) and 25^th ^and 75^th ^percentiles (shaded area) of the reference group on logarithmic Y-axes. Significant difference against the reference group at the 0.001 (***), 0.01 (**) and 0.05 (*) levels after Bonferroni correction is indicated.

After knee injury involving either a MEN alone (Figures 4a,b), or an ACL injury with or without associated MEN (Figures 4c,d), the SF levels of both sGAG and ARGS fragments were elevated within the first four weeks compared with REF (*P *< 0.001). Notably, the median elevations for MEN and ACL patients were more than 200-fold compared with REF for ARGS, but only two- to three-fold for sGAG. For time spans more than four weeks after injury, the sGAG levels of the MEN and ACL groups were not different from the REF group, whereas the ARGS levels continued to differ from REF (except for 26 to 52 weeks after meniscal injury). At none of the time intervals were there any differences between MEN and ACL groups for ARGS or sGAG in SF.

### Proportion of aggrecan detected as ARGS neoepitope in study groups

**Figure 5:**
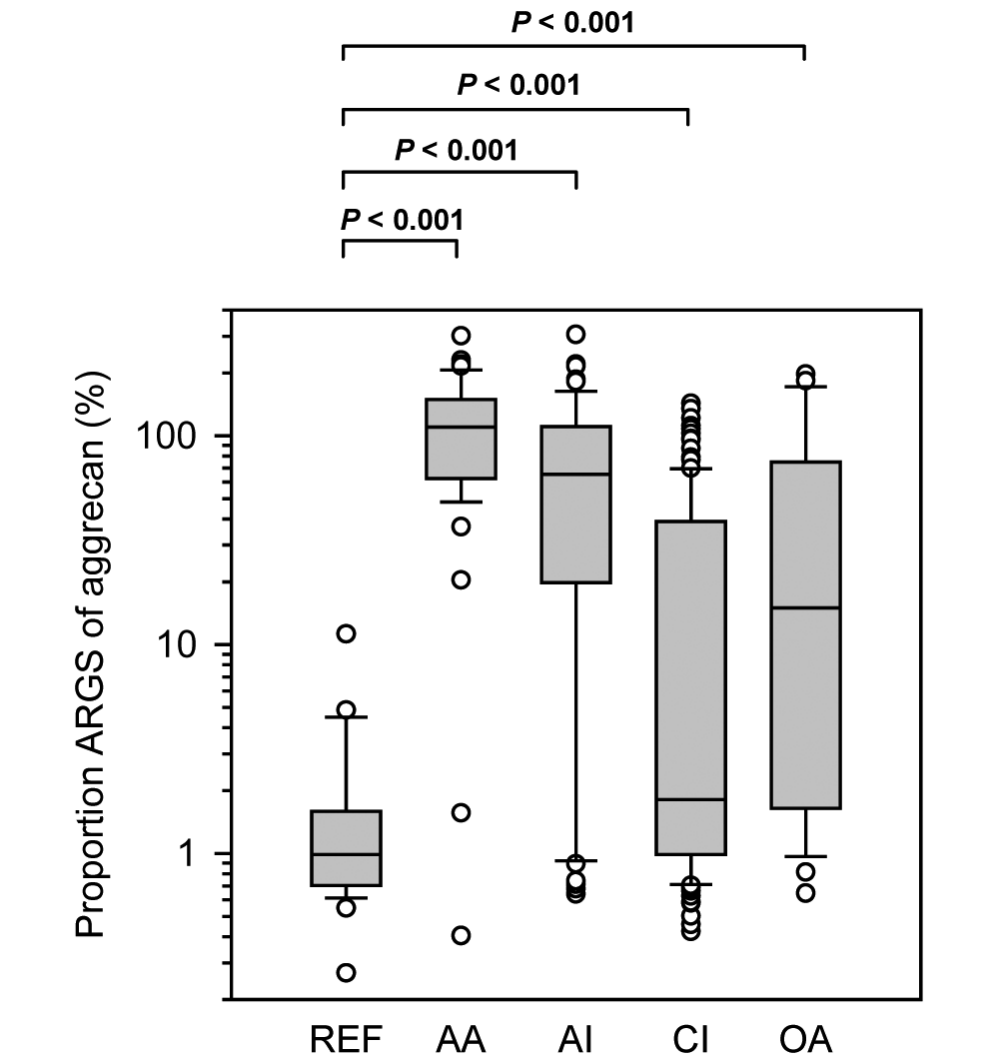
Proportion ARGS of aggrecan in the study groups. The molar proportion of aggrecan fragments in synovial fluid (SF; measured by Alcian blue precipitation) detected as ARGS neoepitope fragments (measured by ARGS ELISA) in the study groups healthy knee reference (REF), acute inflammatory arthritis (AA), acute knee injury (AI), chronic knee injury (CI), and knee osteoarthritis (OA). The boxes define the 25^th ^and 75^th ^percentiles with a line at the median, error bars defining the 10^th ^and 90^th ^percentiles and circles represents individual outliers. After Bonferroni correction, *P *values below 0.013 are considered significant to retain the 0.05 overall significance level. Conversion from microgram sulfated glycosaminoglycan (sGAG)/ml to pmol aggrecan/ml was made assuming an average aggrecan molecular weight of 1.5 × 10^6 ^g/mol and that 75% of this weight was sGAG.

The proportions of aggrecan fragments in SF detected as ARGS neoepitope fragments out of all SF proteoglycan (measured by Alcian blue precipitation) were increased in all study groups compared with REF (*P *< 0.001; Figure 5). The proportion in the AA group was the highest, 111-fold elevated compared with REF, and in the CI group the least increased, two-fold compared with the REF group. The median proportion of ARGS in REF was 1% and ranged in the diagnostic groups from 2% (CI) to 110% (AA). The proportion of ARGS of all aggrecan as a marker for joint disease had a sensitivity of 65% and a specificity of 96% (Table 4).

## Discussion

Aggrecanase cleavage at the Glu-Ala bond in the IGD is important both in animal [6,7,10,11,13,14] and human [4,9,15,17] joint disease. However, previous analyses of larger series of human samples of serum or SF used assays that were not specific for aggrecan fragments carrying this specific neoepitope [1,3,24,26,29-32]. This limits our ability to interpret the results in terms of activity of specific proteases. The work presented here confirms that aggrecanase cleavage in the IGD is a major contributor to aggrecan degradation in human joint pathology, and extends our understanding of the relative contribution of aggrecanase activity in different human joint diseases. We found greatly increased SF concentrations of aggrecan ARGS fragments in several different joint diseases compared with the healthy knee reference group, differences that were only to a small extent reflected by enhanced concentrations of aggrecan fragments in general or sulfated glycosaminoglycans. We also found that the elevation in SF ARGS concentration was most dramatic early after a knee injury, and then decreased to lower levels 12 weeks after the injury, albeit still significantly different from the healthy knee reference group. This suggests that the enhanced aggrecan cleavage in the IGD by aggrecanase caused by the acute joint insult remains increased for several years. Similar long-term changes after knee injury in SF levels of stromelysin (MMP-3) have been reported [2,28].

### Study design and methodology

The range of ARGS concentrations within each study group was substantial, with the exception of the healthy knee reference group. In part, this can be explained by the cross-sectional study design, with the grouping together of individuals with varying severity of injury and disease activity. However, it is also known that the variability of SF markers is greater than for serum and urine markers [32]. Despite the considerable range observed, we note that all study groups differed significantly from the reference group regarding ARGS concentrations. Based on previous studies it is most likely that the knee injury groups are not homogenous regarding progression of OA, but are comprised of progressors and non-progressors [33,34]. It is plausible that heterogeneities like these also influence the ARGS concentrations, and could partly explain the variations seen in these groups.

The lower limit of linearity of the ARGS ELISA in SF was 1 pmol/ml SF, and samples below this level were assigned half that value to allow statistical analysis. All study groups had significantly lower proportions of samples below the lower limit of detection compared with the knee healthy reference group.

As a validation of the ARGS ELISA, we analyzed a subset of SF samples, purified by dissociative cesium chloride density gradient centrifugation, with quantitative western blots using the same ARGS antibody and standard as in the ELISA. The results verified that samples below the detection level of the ARGS ELISA had very low levels of ARGS.

The similarity in the Western blot analysis of loaded and captured aggrecan ARGS fragments show that the ARGS fragments present in the standard and the cesium chloride D1 preparation of an SF sample are captured by the anti-KS plate. The weaker immuno-reaction seen for SF ARGS fragments captured by the ELISA plate, compared with fragments captured from the standard, is most likely a reflection of a lower total ARGS concentration in these SFs compared with the standards.

The strategy applied in the ARGS ELISA of capturing fragments with the anti-KS antibody limits detection of ARGS fragments to those also containing part of the KS domain. Although there are known cleavage sites for proteases such as MMPs, cathepsins, and calpains between ^393^ARGS and the KS domain stretching from amino acid 676 to 848, these cleavage sites were all confirmed to occur by *in vitro *experiments [5]; Sandy and Verscharen showed in SF the presence of a 100 kDa ARGS band ('Species f') estimated to stretch to amino acids 800 to 900 [17]. We detected small amounts of a similar band in SF purified by chromatography or by associative A1 fractioning which, when deglycosylated, migrated to 50 to 70 kDa; the intensity of the band corresponded to about 3% of the total ARGS signal (data not shown). By use of a calculation model [35], we estimate these ^393^ARGS fragments to stretch to amino acids 690 to 750 (data not shown). With the KS domain starting at amino acid 676, these fragments contain part of the domain necessary for capture. We can not, however, completely rule out the presence of SF ARGS fragments not containing the KS necessary for detection.

The inter assay CV for the ARGS ELISA was to a large part caused by the use of two different lot numbers of the KS capture plates supplied by Biosource. The samples were, however, analysed blinded with diagnostic groups spread evenly, so the change of lot numbers had no effect on the observed group differences in ARGS concentrations.

### Aggrecan and ARGS fragments as biomarkers in SF

As reported [1,2,24], the group differences in aggrecan content determined by Alcian blue precipitation or by ELISA with the 1-F21 antibody were small with a maximum of a two-fold increase compared with the REF. Only AA and AI were shown to have significantly elevated levels of sGAG and aggrecan compared with REF.

In contrast to the Alcian blue precipitation method and the 1-F21 ELISA, the ARGS ELISA is highly specific regarding neoepitope and presence of KS on the fragments. Even so, the ARGS neoepitope concentrations correlated with both sGAG and 1-F21 aggrecan concentrations in SF, consistent with previous findings showing that a significant portion of the sGAG and aggrecan content in human SF consisted of neoepitope fragments such as the ARGS fragments measured here, or MMP-generated ^361^FFGV fragments [18,36]. The differences in group median values of ARGS were, however, much greater than for either sGAG or 1-F21 aggrecan. All disease groups were significantly different from the REF, with as much as 177-fold increased levels of ARGS in the AA group. With specificities of 91 to 92%, the concentration of ARGS neoepitope fragments had a sensitivity of 67% in differentiating diseased from healthy patients, compared with sGAG or 1-F21 aggrecan, which had lower sensitivities of 40% and 32%, respectively. Quantification in SF of ARGS fragments generated by aggrecanases by a neoepitope-specific ELISA is clearly a more powerful tool to distinguish diseased and injured joints from healthy than quantification of aggrecan fragments either by 1-F21 ELISA or by measuring sGAG concentrations.

### Proportion of aggrecan detected as ARGS neoepitope

Acknowledging that there are uncertainties in our assumptions of molecular weight and degree of glycosylation of the average aggrecan fragment in SF, and of the molecular weight of the standard, uncertainties that make ARGS proportions of aggrecan greater than 100% possible, the diagnostic groups nevertheless showed large differences in the proportion of SF aggrecan fragments generated by aggrecanase IGD activity. In the two groups most strongly associated with high joint disease activity, acute inflammatory arthritis and acute knee injury, a majority of the aggrecan fragments were indeed shown to be the result of aggrecanase IGD activity, whereas the other groups had much lower proportions. These results corroborate those previously obtained by western blots [4].

### Interpretation of elevated SF levels of ARGS

Based solely on data available in this paper, the elevated SF levels of ARGS in disease, particularly in the acute inflammatory arthritis and acute injury samples, could be explained by enhanced aggrecanase activity against aggrecan resident in the joint cartilage matrix, or against newly synthesized and secreted aggrecan [37]. ADAMTS-5 (aggrecanase-2) was shown to co-localize with hyaluronan surrounding chondrocytes in both normal and osteoarthritic cartilage [38]. However, if enhanced synthesis of aggrecan in combination with aggrecanase activity were to explain the enhanced SF levels of ARGS, an equal increase in the SF levels of G3 was to be expected. This is not the case; we have in quantitative western blot analysis of 30 of these samples seen no significant difference in SF levels of G3 of any of the diagnostic groups compared with healthy knee references [4]. We therefore suggest that an increased aggrecanase activity against the IGD domain of resident aggrecan best explains the enhanced SF levels of ARGS seen in these diagnostic groups.

### The source of the aggrecan fragments

SF is more proximate to the location of joint cartilage and aggrecan degradation than serum or urine, and may therefore better reflect local pathologic processes in the joint being studied. The observed group differences are thus likely to reflect differences in local knee joint pathology. The fragments observed in SF originate in a major part from the joint cartilage, while minor proportions may be released from menisci and ligaments [39,40].

## Conclusions

Our findings confirm that aggrecanase cleavage at the ^392^Glu-^393^Ala bond in the IGD of aggrecan is enhanced in joint pathology, most markedly in acute inflammatory arthritis and early after knee injury, but also in knee OA. The enhanced aggrecanase IGD cleavage is detectable by ELISA as ARGS fragments in the SF. We show that measuring SF concentrations of ARGS is more sensitive in distinguishing diseased and injured joints from healthy ones than methods that do not rely on the specific detection of this aggrecan neoepitope. The ARGS ELISA could be used to monitor aggrecanase activity in joint disease, and to monitor the efficacy of interventions to inhibit this protease activity in joint disease or model systems.

## Abbreviations

AA: acute inflammatory arthritis; ACL: anterior cruciate ligament; ADAMTS: a disintegrin and metalloproteinase with thrombospondin motifs; AEBSF: 4-(2-aminoethyl)-benzenesulfonyl fluoride; AI: acute knee injury; BSA: bovine serum albumin; CI: chronic knee injury; CV: coefficient of variation; EACA: 6-aminohexonic acid; EDTA: ethylenediaminetetra acetic acid; ELISA: enzyme-linked immunosorbent assay; H_2_O_2_: hydrogen peroxidase; IGD: interglobular domain; KS: keratan sulfate; mAb: monoclonal antibody; MEN: meniscal injury; MES: 2-(N-morpholino) ethanesulfonic acid; MMP: matrix metalloproteases; NEM: N-ethylmaleimide; OA: osteoarthritis; PBST: phosphate buffered saline with TWEEN; PMSF: phenylmethylsulfonyl fluoride; PVDF: polyvinylidene difluoride; REF: healthy knee reference; SF: synovial fluid; sGAG: sulfated glycosaminoglycan; TMB: tetramethylbenzidine.

## Competing interests

The authors declare that they have no competing interests.

## Authors' contributions

SL participated in the design of the study, carried out the modification of the ARGS ELISA, the acquisition of data and the analysis and interpretation thereof, and was primarily responsible for writing the manuscript. AS contributed in the design of the study, in the modification of the ARGS ELISA, and helped draft the manuscript. LSL participated in the design of the study, collected samples, provided previous assay data, and helped draft the manuscript. All authors read and approved the final manuscript.
